# Concerns of Infertile Women Candidates for Egg Donation: A Qualitative Study

**Published:** 2020-03

**Authors:** Maryam Bagheri, Mina Jafarabadi, Seyedeh Fatemeh Vasegh Rahimparvar, Ahmad Ali Nourbala, Zahra Behboodi Moghadam

**Affiliations:** 1 Department of Midwifery and Reproductive Health, School of Nursing and Midwifery, Tehran University of Medical Sciences, Tehran, Iran; 2 Vali-e-Asr Reproductive Health Research Center, Tehran University of Medical Sciences, Tehran, Iran; 3 Department of Psychology, School of Medicine, Imam Khomeini Hospital Complex, Tehran University of Medical Sciences, Tehran, Iran

**Keywords:** Infertility, Egg Receiver, Egg Donation, Concern, Reproductive Health, Qualitative Study

## Abstract

**Objective:** Women with premature ovarian insufficiency, menopause, gonadal dysgenesis, and genetic disorders as well as those with a history of recurrent in vitro fertilization failure may benefit from the assisted fertility techniques. These women experience concerns that directly affect their married life especially in egg donation treatment. This study was conducted to investigate the reproductive health concerns of infertile women who were candidates for egg donation.

**Materials and methods:** This qualitative content analysis was conducted in 17 infertile women who were referred to Imam Khomeini Hospital Infertility Clinic from July 2018 to March 2019. The participants were selected through purposeful sampling. Data were collected through individual in-depth semi-structured interviews. A conventional qualitative content analysis approach was adopted for data analysis using the MAXQDA12 software.

**Results:** The results of data analysis showed five themes, including threatened married life, lack of supportive situation, religious beliefs, psychosocial damage, and damaged feminine identity.

**Conclusion:** Infertility is usually accompanied by a great psychological sadness referred to as "stress of infertility". Assisted reproductive techniques (ART) are also a source of stress for patients; therefore, it has a significant impact on the marital relationship and mental health of infertile women. Hence, there is a need for psychological support from the treatment team to reduce woman reproductive health concerns.

## Introduction

Women comprise about half of the world's population and are one of the most important and powerful sources of human and economic power (1). Therefore, any alteration in their health and life situation can affect all aspects of development. The first consequence of the women's health is family dynamism and community growth. A healthy life is the right of all people. To achieve and maintain good health, it is essential to be aware of the factors affecting it (2). One of the most important factors in this regard is reproductive health, which is defined as a state of complete physical, mental and social well-being and not merely the absence of disease or infirmity in all matters relating to the reproductive system and to its functions and processes (3). Diagnosis of infertility is related to increased risk of raising several chronic health conditions in the years following an infertility assessment (4).

According to the International Conference on Population and Development held in Cairo, infertility is considered as one of the aspects of reproductive health that can affect people's health and lives (5). Infertility is one of the major causes of life tension leading to serious psychological, social, emotional, and anxious experiences and even divorce (6, 7). According to several studies across the world, women tolerate the main burden of infertility (8) and infertility causes discord, marital conflicts, violence, stigma, and exclusion and isolation of women (9, 10). Therefore, disrupted couple relationships (11), unsuccessful childbearing attempts, psychological pressure of relatives and friends, and psycho-social factors may affect the reproductive health of infertile couples (12, 13). Moreover, considering egg donation as the only solution for childbearing increases concerns, anxiety, and stress, which have serious impacts on marital relationships (14, 15). Therefore, the sexual and reproductive health of infertile women has always been a challenging subject (16). Several studies have shown that to have a normal pregnancy, the egg recipient should have mental relaxation (17). According to 2014 estimates, there are approximately four million infertile couples in Iran, of whom 5-10% (over 300,000 couples) are candidates for egg or embryo donation methods (18). Women with premature ovarian failure, menopause, gonadal dysgenesis, genetic abnormalities, and a history of recurrent IVF failure can become pregnant if egg donation is used (19). The popularity of egg and embryo donation has increased the demand for these methods in recent years (20). Egg donation has both positive and negative dimensions (21), and lack of a genetic link is one of the most important concerns that infertile couples face (22). 

Considering the concerns of infertile women who are candidates for egg donation should be a health priority. It is necessary to perform studies in this area to provide a solution based on the cultural conditions of the countries to promote reproductive health (23). Therefore, this qualitative study was conducted to identify the reproductive health concerns of infertile women who were candidates for egg donation in order to provide a basis for health policy making and planning in Iran.

## Materials and methods

***Design: ***This qualitative study with a conventional content analysis approach was conducted by the first author. Content analysis is a qualitative descriptive method for the interpretation and classification of textual data through consideration of cultural and contextual aspects influencing the phenomenon under study. The final products of data analysis are categories and themes (24).

***Participants: ***This qualitative study was performed in 17 infertile women that were candidates for oocyte donation (11-13). The subjects were selected from women who were referred to the infertility clinic of Imam Khomeini Hospital affiliated with Tehran University of Medical Sciences. The first author approached eligible participants. Each woman who met the inclusion criteria was provided with information about the study and encouraged to participate in the research. Iranian infertile women of the reproductive age (25) that were candidates for egg donation, had no physical or psychological illness, had a negative history of participation in qualitative interviews about reproductive health, and agreed to express the concerns were included in the study. In order to obtain as many viewpoints as possible, the women who were interviewed were of different demographic backgrounds in terms of age, duration of infertility, education level, and occupation. Only the subjects who met the inclusion criteria and were willing to join the study were included in this research.

***Data collection: ***Data collection was done from July 2018 to March 2019. The interviewer was a PhD candidate in the field of sexual and reproductive health who had practical experience in qualitative research. The eligible women who presented to the infertility clinic to receive infertility treatment were informed of the objectives of the study and invited to take part in in-depth semi-structured interviews. Face-to-face individual interviews were conducted in a private room in the infertility center. The duration of the interview sessions varied from 30 to 90 min with an average of 60 min. Sampling continued until data saturation was achieved and no new data were obtained. The interviews were carried out in Farsi and then translated to English for the purpose of publication of the findings. Each interview was tape-recorded with permission, transcribed verbatim, and analyzed. At the beginning of each interview, the researcher introduced herself and explained the purpose and methodology of the study and then asked the interviewee to sign an informed consent form. Demographic and infertility data were collected using pre-designed questionnaires prior to each interview.

The questions used in the interviews were as follows:

•    Please tell me about your feeling regarding your reproductive health.

•   What were your concerns about your reproductive health after being informed of infertility and the need of egg donation?

•   What are the most important mental concerns when you think about your reproductive health?

In addition, probing questions such as "What do you mean?” or "could you please explain more" were also used in the interviews. 

***Data analysis: ***The data were analyzed based on the method suggested by Graneheim and Landman (24). After each interview, it was transcribed verbatim and read several times to obtain a sense of the whole. The transcripts were then divided into meaning units, which were condensed and labeled with codes. The codes were sorted and divided into subcategories and categories according to their similarities and differences and the hidden contents were developed as themes. The MAXQDA10 software was used for data management. The first author performed the data analysis and the co-authors supervised this process. Any disagreement was resolved through discussion. 

***Trustworthiness:*** Graneheim and Lundman explained that credibility, reliability and data transmission abilities improved the accuracy of qualitative research (24). In this study, purposeful sampling with maximum variation, immersion in data, member checking, and an audit trail were used to validate the coding process and data analysis. Peer checking, long-term engagement with participants, and maintaining ongoing relationships using notes and journals improved the depth of the data analysis.

***Ethical considerations: ***This study was part of the first author’s doctoral dissertation. The study protocol was approved by the Ethics Committee affiliated with Tehran University of Medical Sciences (IR.TUMS.FNM.REC.1397.040). The objective and method of the study were explained to the participants and their permission was obtained for tape-recording the interviews. All participants signed written informed consent forms before the interviews. The participants were assured that the study results would be completely confidential and anonymous, and were informed of the voluntary nature of participation in this study. The subjects had the right to withdraw from the interviews at any time without being penalized. 

## Results

In this study, 17 infertile women aged 29-50 years (mean age = 37±8 years) who were candidates for egg donation were interviewed. The duration of infertility was 3 to 19 years (mean = 5.4 ± 3 years) Eleven (65%) subjects were from different geographical areas of Tehran and 6 (35%) were from other cities. About 41% of the participants were housewives and 59% were employed. In terms of education level, one of the women (5.8%) had unfinished high school education, six (35.2%) had an associate degree or less education, and ten (59%) had a bachelor's degree or higher education (Table 1). 

**Table 1:** Characteristics of participants

**Characteristics**	**Number (%)**
Age	
28-37	8 (47.0)
38-27	8 (47.0)
≤ 47	1 (6.0)
Duration of infertility (Year)	
2-5	11 (65.0)
6-9	3 (17.5)
≤10	3 (17.5)
Educational level of participants	
Junior high school	6 (35.0)
Undergraduate	8 (47.5)
Postgraduate	3 (17.5)
Occupation	
Housewife	7 (41.2)
practitioner	10 (58.8)

A total of 705 codes were extracted from the interviews, which were compared based on content affinity. Data analysis led to the development of 5 themes and 13 subthemes. The themes were “threatened married life”, “lack of supportive situation”, “religious beliefs”, “psychosocial damage”, and the “damaged feminine identity” (Table 2).

**Threatened married life**

Sexual relationship has an undeniable impact on marriage and its cohesion and sustainability. It also has a constructive, important, essential role in mental health. Because of this remarkable and significant characteristic of the sexual desire, it is different from other biological needs and is considered a psychological requirement.

In this study, “threatened marital life” emerged as one of the main themes with the highest frequency, including two subthemes of “damaged sexual relationship” and “husband’s sexual dissatisfaction”.

**Table 2:** Themes and subthemes developed during the data analysis

**Theme**	**Subthemes**
Threatened married life	Damaged sexual relationship
	Husband’s sexual dissatisfaction
Lack of supportive situation	Lack of family support
	Lack of socio-economic support
	Scattered information support
Lack of supportive situation	Trust in God
	Confusion in religious matters
Psychosocial damage	Vague future
	Dual emotions
	Social isolation
Damaged feminine identity	Negative mental image of self
	Threat to feminine identity

***Damaged sexual relationship***: Was one of the identified subthemes. The majority of the participants believed that their sexual relationship was affected by infertility, especially egg donation. They compared sexual experiences before and after the diagnosis of infertility and believed their sexual desire decreased sharply.

*“I had an enjoyable sex before, but this issue, egg donation, has shattered my mind and spirit and distracted me. This is why I am not interested in sex anymore and think this relationship is futile”* (P8, 47 years old).

One of the participant indicated that she faked orgasms after being informed of her infertility. Sex became different to her and she started to pretend enjoying sex, *“After realizing that I have no chance of pregnancy, it seems things have changed, I don't enjoy sex right now, but I pretend to be happy and enjoy it. However, I don't know how long I can pretend”* (P1, 39 years old).

Some of the subjects believed that their sexual satisfaction decreased due to physical problems caused by ovarian dysfunction. *“Not only my sexual desire has decreased but also I feel a burning sensation during sex. I do not enjoy sex, and my husband is unhappy about it. This issue has caused conflicts and problems. My husband says we are not partners anymore; we are like brother and sister”* (P8, 47 years old).

The participants mentioned that scheduled intercourses and medical interventions severely disrupted their sexual function and turned their relationships into mechanical ones because they paid more attention to pregnancy than to sex. *“I really hate that I should have scheduled sex. It is like doing homework or solving tasks. Planned sex is awful and boring”* (P14, 38 years old).

***Husband’s sexual dissatisfaction:*** Was another subtheme of threatened married life. The interviewees believed that their husbands’ sexual attitude had changed. One of the participants stated that despite the enjoyable sex she had with her husband until a few years ago, he sees sex as a way to continue the generation, which is a threat to their life.

*“In the first years of marriage, our sexual relationship was warm, and we were very happy. Now my husband keeps saying why we should have sex when it does not result in a pregnancy. His sexual desire and satisfaction have decreased. If we have sex after weeks, my husband says it is only an instinctive need”* (P16, 44 years old).

This study found that some participants, despite their desire to have sex, were neglected by their husbands, which may be rooted in the husband's dissatisfaction with sex. *“Sometimes, despite my request for sex, my husband refuses and says he is tired, sleepy, or busy. I am worried; this issue worsens our emotional relationship”* (P4, 28 years old).

The present study suggests that infertility can create sexual dysfunction and critical conditions by decreasing the sense of sexual self-efficacy and sexual self-esteem.

**Lack of supportive situation**

Another major theme was lack of supportive situation, which included three subthemes as lack of family support, lack of socio-economic support, and scattered information support. Infertility treatment is sometimes accompanied by an awful pressure that an infertile woman must endure and tolerate. What harms these women even more is the lack of protective mechanisms. This theme had three subthemes, including lack of family support, lack of socio-economic support, and scattered information support.

***Lack of family support*** **:** The source of concern in some of the women was lack of or inadequate family support. Despite initial perceptions and public beliefs, the main pressure on infertile women is not related to financial issues and physical violence, rather, it is the verbal pressure and sarcasm from their husbands or other relatives and acquaintances. In fact, these pressures sometimes serve as a prelude for their husbands to threaten women with divorce or even remarriage.

“*My husband was sympathetic the first time I didn't become pregnant with treatment. For the second time, he constantly blamed me for the costs. He says that he is healthy, so why should he be childless”* (P16, 44 years old).

*“I feel like they don't value me. My sister-in-law was pregnant, but they did not tell me until delivery, because they thought I was jealous. This behavior makes me sad. They do not tell me directly, but their manners are offensive”* (P10, 29 years old).

Thus, most of the participants in the present study suffered from families that not only did not have a supportive role, but also were often the cause of pressure.

***Lack of socio-economic support***: The participants believed that there was lack of financial support, poor coverage of treatment costs, lack of ministry-approved centers to find donors, and lack of serious efforts by the treatment team to reduce patient suffering.

*“I applied for a job due to the high costs of egg donation, because I didn't want my husband or my parents to pay for me. Unfortunately, insurance does not cover the costs of the donation. It seems we do not have any rights to have children; moreover, private hospitals are very expensive”* (P7, 44 years old).

Most infertile women tended to hide the use of donation treatment; therefore, many of them experienced opportunistic and dishonest donors.

***Scattered information support***: According to the participants, not enough attention has been paid to public information on egg donation. For instance, the mass media do not play an active role in this regard.

*“When there are no TV programs about donation, we can't expect people around us to understand either. In other countries, experts make movies and TV series about these subjects; therefore, people are also aware of this issue”* (P3, 30 years old).

In this study, some the participants’ concerns included lack of access to accurate and complete information about the donors' moral, physical, and mental health as claimed by donors.

“*It is important for me that the donor is smart and has a healthy condition physically and mentally. I only see her appearance. I think doctors should check. The donors are unreliable”* (P11, 35 years old).

**Religious beliefs**

One of the main themes that emerged from the present study was religious beliefs. In many societies such as Iran, religious values affect the people’s behaviors. The present study emphasized the presence of strong religious beliefs in the majority of participants, which could help them to achieve the goal of childbearing. This theme had two subthemes, including trust in God and confusion in religious matters.

***Trust in God*** **: **The participants were waiting for a miracle from God to become pregnant and believed that infertility was a blessing in disguise for them. “*My husband always prays to have children, and says miracle is for us like Prophet Abraham. He thinks prayers will change our destiny and we will finally have a baby”* (P2, 35years old).

***Confusion in religious matters*** **: **Generally, in the Shia jurisprudence, the consensus of Maraji (despite divergent views on donation) is that the practice of egg donation is religious. In the present study, almost all participants disagreed with a marriage contract between donors and their spouses and believed that it endangered their married life.

*“We went to the center a few years ago. They said my husband had to marry that woman, but I disagreed. You know it is a feminine feeling. I'm sure of my husband; however, that woman may not let go”* (P9, 49 years old).

**Psychosocial damage**

Psychosocial damage was another major theme. Infertility provided certain conditions leading the participants to social isolation, which worsens their reproductive health problems. This theme had three subthemes, including vague future, dual emotions, and social isolation.

***Vague future*** **:** The participants stated that their fertility, future, and married life were vague and their future was not clear and transparent. *“When I heard the only way to treat infertility was to use egg donation, I was really scared. I thought about a hundred things at the time, for example, do I get pregnant? What will happen to my married life without donation or with a child born to egg donation? I am totally disappointed”* (P13, 32 years old).

Most of the participants tended to hide the use of egg donation from people around them. Concerns about revealing the use of this treatment option and the unpredictability of the child's reaction preoccupied them. They believed that if they disclosed the use of egg donation, their life would be like a movie.

“*I'm worried that if my child learns about egg donation, like what we see in the movies, the kid will say you are not my mother and leave us, so this is very distressing. It is not fair; I suffer, but he forgets everything when he grows up. That's why I disagree with disclosure of egg donation, because we will lose peace in our life”* (P5, 41 years old).

Another concern of the participants was the fear of potential harassment of donors in the future. Almost all of the participants were had this concern. At the same time, they were also worried about the idea of a childless future.

*“An infertile woman thinks about many things. There are a lot of concerns. I cannot predict my life in the future. My future without kids is certainly vague and dark. The future must be brighter with the kids”* (P11, 35 years old).

***Dual emotions*** **: **In the present study, the participants were hopeless and hopeful. They were optimistic about the outcome on the one hand but they could not have a positive prediction on the other hand. Many contradictory remarks were expressed by the participants.

“*It was very difficult to accept the egg donation. I felt like I reached a dead end. I don't know how to put it into words; it was a very bad feeling, I felt like my heart was empty, a kind of sheer frustration. However, I was also hopeful. I thought about becoming a mother and hugging my baby, something like suspension between hope and failure”* (P13, 32 years old).

***Social isolation*** **:** Social isolation was another subtheme of psychosocial damage. The participants believed that infertility led to social changes and isolation in their social activities. They were sometimes forced to leave or change their jobs due to constant questions of others and their inappropriate behavior*. “You cannot sit with friends and family; you cannot chat with anyone. They judge you; because they have children, they think you are jealous of them. I don't have any family relationships right now; I do not feel comfortable in family and friendly parties, my heart aches”* (P4, 28 years old).

**Damaged feminine identity**

Damaged feminine identity emerged as one of the main themes. This theme had two subthemes, including negative mental image of self and threat to feminine identity.

***Negative mental image of self*** **:** The results showed that most of the participants considered infertility as a major weakness. They believed that this disadvantage prevented them from making comments on the most obvious issues of everyday life. *“You can't even comment in the family anymore because you are infertile. If you want to talk about raising a child, they say you do not understand because you do not have one. If we even talk about financial problems, they wonder if we have any expenses at all! It seems infertility is undermining our ability to understand everyday issues” *(P14, 43 years old).

Lack of companionship, especially their spouses, made these women feel negative about themselves. These feelings led to sadness and depression.

***Threat to feminine identity:*** In fact, lack of children deprived the woman from being a mother. They were of the opinion that using egg donation, even if it ended in pregnancy, would confirm their inability. “*Using egg donation indicates that I am incapable. It provides evidence for my weakness, even after the birth of the baby. I will always remember that I was not able to have a baby with my own genes”* (P17, 48 years old).

The participants felt inferior because of the negative attitude of the community and their spouses. One of the participants expressed said, *“I felt so bad. I thought it was impossible to have a baby. I was very upset and felt inferior. I felt ashamed in front of my husband, family members and friends. I wondered what others thought about me”* (P14, 38 years old).

At the end of the interview, the researcher asked the participants which of the concerns were the most important to them. Four participants stated that parenting was the most important priority in their lives. Other participants believed that if their sexual relationship improved, the risk of losing their spouse would reduce and on the other hand, their chances of childbearing would increase by changing the treatment process.

## Discussion

This was the first qualitative study of the concerns of infertile women candidates for egg donation in an Iranian setting. In this study, the concerns were highly correlated and overlapping and sometimes one concern concealed other worries within itself. Moreover, one concern could cause other concerns for the patient. For instance, concerns about the low chance of pregnancy with egg donation led to the fear of divorce, which ultimately resulted in reduced marital satisfaction. The results of this study showed that sexual relationship was the most important concern of almost all participants. They believed that their sexual relationship was affected by their infertility. Other important concerns were lack of supportive situation, religious beliefs, psychosocial damage, and damaged feminine identity. 

Studies investigating the impact of infertility on sexual relationship and married life have shown contradictory results (26). Oddens et al (27), Coeffin-Driol (28), Muller (29), and Repokari (30) found that infertility and its treatment did not have a negative impact on the sexual function and sexual satisfaction of infertile couples. They also showed that shared stress resulting from a common problem brought the infertile couple closer together and improved the relationship between couples (26-30). The results of the above studies are not consistent with the results of the present study.

Reproductive health and sexual function can be a source of concern for people (31). Several investigations including studies conducted by Piva (32) and Martins (33) found that sexual dysfunction might occur following the diagnosis and treatment of infertility and could cause profound marital and emotional problems (32, 33). Infertility has negative impacts on the marital relationships and leads to unpleasant sexual activity (34-41). Aduloju found that the quality of life of infertile women, especially their own and their partners’ marital status, was lower than a control group (42). A review study by Lara revealed that sexual and marital satisfaction decreased after a diagnosis of infertility and during the treatment process (43). The results of the above studies are in line with the findings of the present study.

The most common sexual dysfunction in infertile women is decreased libido and lack of orgasm (35, 44). It seems that planned intercourses based on infertility treatment severely disrupts the women's sexual function and makes the couples’ relationships unpleasant (45). Thus, the couples’ unfavorable relationships, failed attempts at childbearing, and the stressors of those around them may alter their sexual function (46, 47). The inconsistency in the results of studies in this field may be due to factors affecting sexual function such as age, length of marriage, spouse’s age, education level, income level, duration of infertility, gender, type of infertility, and body mass index (48, 49, and 50). The above factors were not addressed in this study, so further quantitative studies are required in this regard. 

The results of the present study showed that another cause of concern for the participants was the lack of supportive situation and unawareness about the donation process in the society. A study by Purewal (51) indicated that awareness of gamete donation methods was very low and people did not have a respectable attitude toward using donation techniques. Moreover, the results of this study also showed that the society had a negative approach towards infertile couples (51). Lack of awareness, negative attitudes towards gamete donation, and the stigma of donation methods result in a negative attitude towards the donors as well (52). Improving public awareness and knowledge about this treatment modality reduces the fear and stigma of donation; furthermore, the public will view it as a humanitarian act like organ donation (53). It seems that mass media should take measures to improve the public awareness of donation methods in the community. In the present study, the participants also expressed concerns about the lack of physical resemblance to the offspring. Other concerns of the subjects were donation costs, lack of supervision on infertility centers, and lack of insurance facilities. Similar to our findings, a study by Greenfield in the US also found that donor selection depended on factors such as financial issues, availability, and concerns about donor motivation and characteristics (54). In many societies, religious values are one of the factors affecting the people’s behavior and choice of treatment (55).

The present study emphasized the power of religious beliefs in an overwhelming majority of the participants that, despite infertility, could be helpful in achieving pregnancy. A study by Izadyar showed that many factors, such as the power of religious beliefs and religious permission, played an important role in the acceptance of the egg donation method (56). Latif Nejad found that most religious infertile women tried to evaluate infertility in a spiritual way and their treatment choices were influenced by their religious views (57). Inhorn revealed that in Muslim countries, religion has a great influence on the choice of treatment (55). A study by Razzaghi showed that infertile women followed religious leaders in choosing gamete and embryo donation methods; in addition, their awareness of the legitimacy of these methods had a profound effect on their application (58). In a study by Ramezanzadeh, the majority of the egg recipients did not view donation treatment as ethically and religiously approved although they considered it as the last solution (59). 

However, the results of the present study showed that despite the power of religious beliefs to continue treatment, confusion in religious matters was seen in the participants. The participants of this study preferred married life to other issues. Almost all of the participants disagreed with the idea of a marriage contract between donors and their spouses as they believed it endangered their married life, which may be due to the role of the current culture in the society at this time.

Psychosocial complications of infertility are associated with stress and social maladjustment (60) and depend on factors such as the duration of infertility, history of previous failed treatments (61), age, and cultural, social, and ethnic background (62). A study by Inhorn in the US found that the stigma of infertility could lead to psychological trauma (63). An international study conducted in Belgium, France, and the Netherlands highlighted the role of infertility as a stressor among stressful life experiences (64). Infertility leads to insecurity in married life and social stigma in developing societies (65). One of the concerns of the infertile women in this study was the tendency to hide the use of egg donation. In this regards, Hadizadeh found that egg recipients were willing to hide the use of egg donation to avoid stigma and other people’s judgment (66). The results of the present study were consistent with other studies in this area and psychological counseling seems to be necessary for infertile women, especially in cases of egg donation, to resolve their concerns (67).

The infertility and the resulting anxiety are associated with adverse effects on the self-esteem, sexual identity, self-confidence, and body image, which inevitably affect the couples' sexual relationship, desire, and satisfaction (68). Infertility and its treatment result in a feeling of guilt and reduced self-esteem (69, 70). Infertility may be considered a disease in many cultures; however, the results of the present study showed that not only it was viewed as a failure in the family formation process, but also it was associated with identity loss in woman and prevented them from achieving their aspirations in the Iranian culture. Therefore, infertile people experience high levels of anxiety and physical symptoms due to exposure to difficult and painful treatments, long waiting periods, loneliness, rejection, and fear of treatment failure (71). Considering egg donation as the only solution for pregnancy increases the levels of stress and anxiety in infertile women and severely affects their marital relationships (72, 73). Several studies have shown that it is important to identify the concerns of infertile women who are candidates for egg donation during the difficult decision-making period for accepting donation treatment. Due to the increasing number of infertile women who are candidates for egg donation (18), it seems that more attention should be paid to their reproductive health concerns.

In Iran, despite the widespread use of egg donation to treat infertile women, the reproductive health and sex life of these women have received little attention from experts and policymakers (68). It is important to identify the concerns of these women, which may have serious consequences on their reproductive health, to design proper interventions (74). Therefore, it is necessary to address reproductive health concerns and needs of the infertile women who are candidates for oocyte donation using modern and valid Persian questionnaires (75) and counseling (76).

***Limitation: ***The sensitivity of the research topic, the shame associated with issues related to the reproductive system and sexual intercourse, and the inability of the participants to express the reality in words were some limitations of the present study. Another limitation was that the husbands of the infertile women were not assessed. Some of the limitations of the present study were overcome through explaining the importance of the research, communicating with participant and adhering to ethical principles in order to gain the trust and confidence of the participants. 

***Suggestions:*** It is suggested that qualitative studies be conducted on other methods of donation, including sperm donation, embryo donation, and surrogacy, to identify the concerns and provide guidelines.

## Conclusion

Infertility is often accompanied by a psychological sadness resulting from the "stress of infertility" (77). Assisted reproductive techniques are also a source of stress for patients; therefore, to reduce sexual dysfunction, it is important that the patients receive psychological support from the treatment team. Psychological services should be provided by trained infertility counselors (19). Providing sufficient information and attitude change are important factors in reducing psychological disorders (78). 

One of the problems of the Iranian society is lack of information and awareness about infertility, especially gamete donation, and incorrect attitudes and beliefs about this treatment option. Lack of proper information and inadequate education about sexual activity, incorrect sexual views, and anxiety about sexual function all contribute to the development and persistence of sexual disorders (64). Therefore, it seems that more attention should be paid to psychosexual counseling for women who are candidates for fertilization.

## References

[1] Harlow SD, Campbell OM (2004). Epidemiology of menstrual disorders in developing countries: a systematic review. BJOG..

[2] 29th United Nations General Assembly Special Session on the follow-up to the Programme of Action of the International Conference on Population and Development Beyond 2014.

[3] Zegers-Hochschild F, Adamson GD, de Mouzon J, Ishihara O, Mansour R, Nygren K (2009). The International Committee for Monitoring Assisted Reproductive Technology (ICMART) and the World Health Organization (WHO) revised glossary on ART terminology, 2009. Human Reproduction..

[4] Murugappan G, Li S, Lathi RB, Baker VL, Eisenberg ML (2018). Increased risk of incident chronic disease in infertile women: analysis of US claims data. Fertility and Sterility.

[5] Cairo, Egypt (1994).

[6] Rooney kl, Domar AD (2018). The relationship between stress and infertility. Dialogues Clin Neurosci..

[7] Berek JS, Novak E (2012). Berek & Novak's gynecology.

[8] Abasi Shavazi MJ, Asgai Khaneghah A, Razeghi Nasrabad HB (2005). Life Experience with Infertility; a case study in Tehran. Women research. J ReProd Infertil..

[9] World Health Organization (2001). Current practices and controversies in assisted reproduction. Sexual and reproductive health. Report of a meeting on “Medical, Ethical and Social Aspects of Assisted Reproduction” held at WHO Headquarters in Geneva, Switzerland.

[10] Bokaie M, Simbar M, Yassini Ardekani SM (2015). Sexual behavior of infertile women: a qualitative study. Iran J Reprod Med.

[11] Nelson CJ, Shindel AW, Naughton CK, Ohebshalom M, Mulhall JP (2008). Prevalence and predictors of sexual problems, relationship stress, and depression in female partners of infertile couples. J Sex Med.

[12] Perlis N, Lo KC, Grober ED, Spencer L, Jarvi K (2013). Coital frequency and infertility: Which male factors predict less frequent coitus among infertile couples?. Fertil Steril.

[13] Dumont F (2010). A History of Personality Psychology: Theory, Science, and Research from Hellenism to the twenty–First Century.

[14] Marci R, Graziano A, Piva I, Lo Monte G, Soave I, Giugliano E (2012). Procreative sex in infertile couples: the decay of pleasure?. Health and Quality of Life Outcomes.

[15] Wischmann TH (2013). Sexual disorders in infertile couples: an update. Curr Opin Obstet Gynecol.

[16] Vakilian K, Mirzaii K, NajmAbadi . (2011). Reproductive health in Iran: international conference on population and development goals. Oman Med J.

[17] Bracewell-Milnes T, Saso S, Abdalla H, Thum MY (2018). A systematic review investigating psychosocial aspects of egg sharing in the United Kingdom and their Potential effects on egg donation numbers. Hum Fertil (Camb).

[18] Direkvand Moghadam A, Delpisheh A, Sayehmiri K (2014). The trend of infertility in Iran, an original review and meta-analysis. Nurs Pract Today.

[19] Gardner DK, Weissman A, Howles CM, Shoham Z (2018). Textbook of Assisted Reproductive techniques Technologies: Laboratory and Clinical Perspectives (Reproductive Medicine and Asst. Reproduction).

[20] (2015). National Centers for Chronic Prevention and Health Promotion division of Reproductive Health Assisted Reproductive Technology.

[21] Golombok S, Cook R, Bish A, Murray C (1995). Families Created by the New Reproductive Technologies: Quality of Parenting and Social and Emotional Development of the Children. Child Dev.

[22] Bustillo M, Krysa LW, Coulam CB (1995). Uterine receptivity in an oocyte donation programme. Hum Reprod.

[23] Wischmann T, Schilling K, Toth B, Rosner S, Strowitzki T, Wohlfarth K (2014). Sexuality, self-esteem and partnership quality in infertile women and men. Geburtshilfe Frauenheilkd.

[24] Graneheim UH, Lundman B (2004). Qualitative content analysis in nursing research: concepts, procedures and measures to achieve trustworthiness. Nurse Educ Today.

[25] Inhorn MC (2009). Right to assisted reproductive technology: overcoming infertility in low-resource countries. Int J Gynaecol Obstet.

[26] Jain K, Radhakrishnan G, Agrawal P (2000). Infertility and psychosexual disorders: Relationship in infertile couples. Indian J Med Sci.

[27] Oddens BJ, den Tonkelaar I, Nieuwenhuyse H (1999). Psychosocial experiences in women facing fertility problems a comparative survey. Hum Reprod.

[28] Coeffin-Driol C, Giami A (2004). The impact of infertility and its treatment on sexual life and marital relationships: review of the literature. Gynecol Obstet Fertil.

[29] Muller MJ, Schilling G, Haidl G (1999). Sexual satisfaction in male infertility. Arch Androl.

[30] Repokari L, Punamaki RL, Unkila-Kallio L, Vilska S, Poikkeus P, Sinkkonen J (2007). Infertility treatment and marital relationships: a 1-year prospective study among successfully treated ART couples and their controls. Hum Reprod.

[31] Direkvand Moghadam A, Delpisheh A, Direkvand Moghadam AZ (2015). Effect of Infertility on Sexual Function: A Cross-Sectional Study. J Clin Diagn Res.

[32] Piva I, Lo Monte G, Graziano A, Marci R (2014). A literature review on the relationship between infertility and sexual dysfunction: does fun end with baby making?. Eur J Contracept Reprod Health Care.

[33] Martins MV, Peterson BD, Almeida V, Mesquita-Guimaraes J, Costa ME (2014). Dyadic dynamics of perceived social support in couples facing infertility. Hum Reprod.

[34] Tao P, Coates R, Maycock B (2011). The impact of infertility on sexuality: A literature review. Australas Med J..

[35] Klemetti R, Raitanen J, Sihvo S, Saarni S, Koponen P (2010). Infertility, mental disorders and well-being--a nationwide survey. Acta Obstet Gynecol Scand.

[36] Jamali S, Zarei H, Rasekh Jahromi A (2014). The relationship between body mass index and sexual function in infertile women: a cress-sectional survey. Iran J Reprod Med.

[37] Bayar U, Basaran M, Atasoy N, Kokturk F, Arikan II, Barut A (2014). Sexual dysfunction in infertile couples: evaluation and treatment of infertility. J Pak Med Assoc.

[38] Bunting L, Boivin J (2008). Knowledge about infertility risk factors, fertility myths and illusory benefits of healthy habits in young people. Hum Reprod.

[39] Schmidt L (2009). Social and psychological consequences of infertility and assisted reproduction - what are the research priorities?. Hum Fertil (Camb).

[40] Aggarwal RS, Mishra V, Jasani AF (2013). Incidence and prevalence of sexual dysfunctionin infertile females. Middle East Fertility Society Journal..

[41] Khodakarami N, Hashemi S, Seddigh S, Hamdiyeh M, Taheripanah R (2010). Life experience with infertility; a phenomenological study. J Reprod Fertil.

[42] Aduloju OP, Olaogun OD, Aduloju T (2018). Quality of life in women of reproductive age: a comparative study of infertile and fertile women in a Nigerian tertiary centre. J Obstet Gynaecol.

[43] Lara L, Salomao PB, Romao AP, Reis RM, Navarro PA, Rosa-e-Silva AC (2015). Effect on infertility on the sexual function of couples: state of the Art. Recent Pat Endocr Metab Immune Drug Discov.

[44] Buster JE (2013). Managing female sexual dysfunction. Fertil Steril.

[45] Zare Z, Amirian M, Golmakani N, Mazlom R, Laal Ahangar M (2016). Sexual dysfunction in infertile women. Int J Reprod BioMed (yazd).

[46] Besharat MA, Hoseinzadeh Bazargani R (2006). A comparative study of fertile and infertile women's mental health and sexual problems. Iranian journal of psychiatry and clinical psychology.

[47] Ozturk S, Sut HK, Kucuk L (2019). Examination of sexual functions and depressive symptoms among infertile and fertile women. Pak J Med Sci.

[48] Audu BM (2002). Sexual dysfunction among infertile Nigerian women. J Obstet Gynaecol.

[49] Farajkhoda T, Latifnejad Roudsari R, Abbasi M (2013). An exploratory study to develop a practical ethical framework for reproductive health research. Iran J Reprod Med.

[50] Kucur Suna K, Ilay G, Aysenur A, Kerem Han G, Eda Ulku U, Pasa U, Fatma C (2016). Effects of Infertility Etiology and Depression on Female Sexual Function. J Sex Marital Ther.

[51] Purewal S, van den Akker OB (2009). Systematic review of oocyte donation: investigating attitudes, motivations and experiences. Hum Reprod Update.

[52] Purewal S, van den Akker OB (2006). British women’s attitudes towards oocyte donation: Ethnic differences and altruism. Patient Educ Couns.

[53] Englert Y (1996). Ethics of oocyte donation are challenged by the health care system. Hum Reprod.

[54] Greenfeld DA, Klock SC (2004). Disclosure decisions among known and anonymous oocyte donation recipients. Fertil Steril.

[55] (2003). Local babies, Global Science: Gender, Religion and in Vitro Fertilization in Egypt.

[56] Bagheri-Lankarani N, Zarei F, Zandi M, Omani Samani R, karimi M (2014). The Experiences of Women Fertilized through Egg Donation during Their Treatment Process. Evidence Based Care Journal.

[57] Latifnejad Roudsari R, Allan HT, Smith PA (2009). Navigating the spiritual journey of infertility: Muslim and Christian infertile women’s experiences. Religion and Psychology. Nova Science Publishers..

[58] Razeghi Nasrabad HB (2005). “Socio-cultural aspect of infertility in Iran.” PhD Thesis.

[59] Ramezanzadeh F, Haghollahi F, Bagheri M, Masoomi M, Abedi-Nia N, Jafar-Abadi M (2009). Attitudes of Donors and ReciPients Toward Ethical Issues in Oocyte Donation. Journal of Reproduction& Infertility..

[60] Eggers S, Kirchengast S (2001). The polycystic ovary syndrome–a medical condition but also an important psychosocial problem. Coll Antropol.

[61] Ridenour AF, Yorgason JB, Peterson B (2009). The Infertility Resilience Model: Assessing Individual, Couple, and External Predictive Factors. Contemporary Family Therapy.

[62] Fekkes M, Buitendijk SE, Verrips GHW, Braat DDM, Brewaeys AMA, Dolfing JG (2003). Health‐related quality of life in relation to gender and age in couples planning IVF treatment. Human Reproduction..

[63] Inhorn MC, Van Balen F (2002). Infertility around the Globe: New Thinking on Childlessness, Gender, and Reproductive Technologies.

[64] Mohammadi MR, Khalajabadi Farahani F (2001). Emotional and Psychological Problems of infertility and strategies to overcome them. Journal of Reproductive& Infertility.

[65] Winkvist A, Akhtar HZ (2000). God should give daughters to rich families only: attitudes towards childbearing among low-income women in Punjab, Pakistan. Soc Sci Med.

[66] Hadizadeh-Talasaz F, Latifnejad Roudsari R, Simbar M (2015). Decision for disclosure: The experiences of Iranian infertile couples undergoing assisted reproductive donation procedures. Hum Fertil (camb).

[67] Soderstrom-Anttila V, Salevaara M, Suikkari AM (2010). Increasing openness in oocyte donation families regarding disclosure over 15 years. Hum Reprod.

[68] Shahraki Z, Davari Tanha F, Ghjarzadeh M (2018). Depression, Sexual dysfunction and sexual quality of life in women with infertility. BMC women’s health.

[69] Shoji M, Hamatani T, Ishikawa S, Kuji N, Ohta H, Matsui H (2014). Sexual Satisfaction of infertile couples assessed using the Golombok-Rust Inventory of Sexual Satisfaction (GRISS). Sci Rep.

[70] Reporaki L, Punamaki RL, Unkila-Kallio L, Vilska S, poikkeus P, Sinkkonen J (2007). Infertility treatment and marital relationship: a 1-year prospective study among successfully treated ART couples and their controls. Hum Reprod.

[71] Kucur Suna K, Ilay G, Aysenur A, Kerem Han G, Eda Ulku U, Pasa U (2016). Effects of Infertility Etiology and Depression on Female Sexual Function. J Sex Marital Ther.

[72] Winkelman WD, Katz PP, Smith JF, Rowen TS, Infertility Outcomes Program Project Group (2016). The sexual impact of infertility among women seeking fertility care. Sex Med.

[73] Zabihi rigcheshme M, Mirzaian B, Hasanzade R, Shahidi M (2012). Comparing sexual attitudes of fertile and infertile couples. J Mazandaran Univ Med Sci.

[74] Chachamovich JR, Chachamovich E, Ezer H, Fleck MP, Knauth D, Passos EP (2010). Investigating quality of life and health-related quality of life in infertility: a systematic review. Journal of Psychosomatic Obstetrics & Gynecology.

[75] Cai L, Liu J, Lu SH, Yin J (2015). Female Sexual Dysfunction and Timed Intercourse: A Prospective Study of 105 infertile women. Advances in Reproductive Sciences.

[76] Hadizadeh-Talasaz F, Simbar M, Esmaily H, Latifnejad Roudsari R (2019). Development and Validation of a Decision-Making Donor Conception Questionnaire in Iranian Infertile Couples. International Journal of Fertility & Sterility.

[77] Latifnejad Roudsari R, Hadizadeh Talasaz F, Simbar M, Khadem Ghaebi N (2014). Challenges of Donor Selection: The Experiences of Iranian Infertile Couples Undergoing Assisted Reproductive Donation Procedures. Iranian Journal of Obstetrics, Gynecology and Infertility.

[78] Pacheco Pahla A, Lourenco MF (2011). Psychological and Cross-Cultural aspects of infertility and human sexuality. Adv Psychosom Med.

